# A protocol for mobilising novel finance models for collaborative health promotion and disease prevention initiatives: taking a smart capacitating investment approach in the Invest4Health project

**DOI:** 10.3389/fpubh.2024.1426863

**Published:** 2025-01-23

**Authors:** Joanna Lane, Rhiannon T. Edwards, Balázs Babarczy, Holly Whiteley, Vidya Oruganti, Maureen Rutten-van Mölken, Caroline Costongs, Anant R. Jani, Sarah Wordsworth, Alison Maassen, Apostolos Tsiachristas, Jacob Davies, Bengt Stavenow, Jolanda van Vliet, Steve Wright, Lina Papartyte, Jennifer Catherine Camaradou, Rositsa Koleva-Kolarova

**Affiliations:** ^1^Stichting Health ClusterNET, Amsterdam, Netherlands; ^2^Centre for Health and Technology, University of South-Eastern Norway (USN), Kongsberg, Norway; ^3^Centre for Health Economics and Medicines Evaluation (CHEME), Bangor University, Bangor, United Kingdom; ^4^Syreon Research Institute, Budapest, Hungary; ^5^NHH Norwegian School of Economics, Bergen, Norway; ^6^SNF, Centre for Applied Research at NHH, Bergen, Norway; ^7^Erasmus School of Health Policy and Management, Erasmus University Rotterdam, Rotterdam, Netherlands; ^8^EuroHealthNet, Brussels, Belgium; ^9^Heidelberg Institute for Global Health, Heidelberg University, Heidelberg, Germany; ^10^Nuffield Department of Population Health, Oxford NIHR Biomedical Research Centre, University of Oxford, Oxford, England, United Kingdom; ^11^Nuffield Department of Primary Care Health Sciences, University of Oxford, Oxford, England, United Kingdom; ^12^Innovation Skåne, Lund, Sweden; ^13^Department of Future Public Health Services, Kristianstad, Sweden; ^14^The Bartlett Faculty of the Built Environment, University College London, London, England, United Kingdom; ^15^St Anne’s College, University of Oxford, Oxford, England, United Kingdom

**Keywords:** health economics, public health, smart capacitating investment, prevention, innovative financing mechanisms

## Abstract

**Background:**

The prevalence of preventable non-communicable disease (NCD) underpins the need for a life-course and cross-sectoral approach to population health that is grounded in health promotion and disease prevention. European Union (EU) countries typically spend 6 to 13% of gross domestic product (GDP) on health care, yet less than 3% of this is dedicated to prevention. The extent to which spending in other sectors prevents avoidable ill-health is largely unknown. The lack of fiscal space post-COVID-19 means shifting from models of care built around treatment to those with greater emphasis on prevention will require innovative, evidence-based investment within and between sectors. The term “smart capacitating investment” (SCI) has previously been used to understand how to best boost social infrastructure investment in education, health, transport and housing across the EU. Here we take that idea further by exploring the applicability of SCI to public health financing to improve population health and well-being.

**Aim:**

To explore and develop innovative SCI models and tools that enable collaboration and investment across health ecosystems for enhanced health promotion and disease prevention, test them in diverse real-world settings, and create a roadmap for large-scale implementation.

**Methods:**

The Invest4Health (I4H) project brings together transdisciplinary expertise in epidemiology, public health, health economics, population science, business management, finance, implementation and social sciences, digital health innovation, and regional health systems. The project consists of eight work packages which span the exploration and conceptualisation of SCI in public health; the characterisation of SCI-compatible business and finance models; piloting and evaluation of these models in four European testbeds (Sweden, Germany, Spain and Wales UK); and exploring the opportunities for sustainable replication and scaling of SCI and future research.

**Discussion:**

We present an introduction to the I4H project, the concept of SCI applied to public health, plus key points for discussion internationally.

## Background

### Health and not just health care

Globally, 48% of non-communicable disease (NCD) burden is attributed to environmental, occupational, metabolic, and behavioural risk factors (1), and the record for reducing exposure to preventable harmful risks over the last three decades has been poor (2). More evidence-based action is necessary to realise the promise of primary and secondary prevention. To reduce the imbalance of funding largely aimed at treatment rather than prevention, a recent Organisation for Economic Co-operation and Development (OECD) report called for an increased proportion of the OECD’s average gross domestic product (GDP) of around 1.4% to be directed to funding primary and secondary prevention, over and above the total health expenditure of around 9% of GDP across OECD countries in 2019 (3). Public investment in health promotion and disease prevention has been under downward pressure ever since the 2008 economic crisis, both at national and subnational level, and significant investment gaps remain despite recent investment to address the COVID-19 crisis (4). To fill these gaps, we need to shift existing resources around and/or bring new investors into the public health ecosystem. Arguably, part of this 1.4% of GDP could be spent by sectors outside the health sector which have a capacity to influence or create the physical and socio-economic environments that can help prevent avoidable ill-health, disability, and premature mortality.

Social determinants and health behaviours, such as diet, physical activity, smoking and alcohol consumption, have an important impact on individual health across different stages of the life-course (5–8). Social determinants are the conditions in which people are born into, grow, work and live in and age into, and the wider set of forces and systems shaping the conditions of daily life (5–8). These are linked to commercial determinants, which include the systems, practices, and pathways through which commercial actors, from small firms to transnational corporations, can directly and indirectly impact health and equity of health, both positively and negatively (9, 10). Gilmore et al. (9), for example, recently highlighted that large multinational corporations in the tobacco, ultra-processed food, and alcohol sectors, which are responsible for at least one third of all global deaths, are directly responsible for increasing avoidable ill-health, especially for the most socially disadvantaged subgroups.

**Figure 1:**
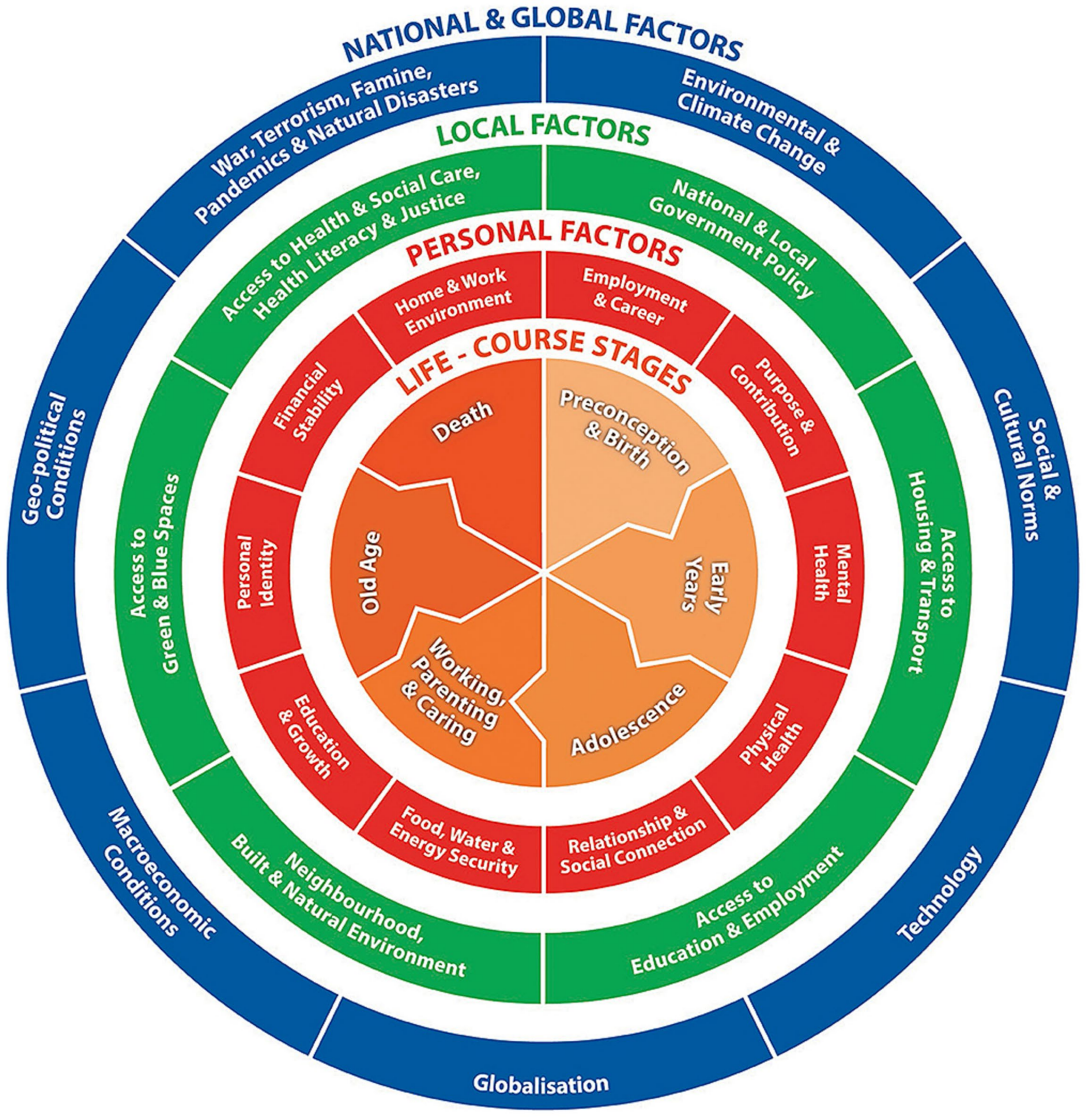
The well-being and well-becoming wheel (6).

Social and commercial determinants are interconnected and can be complementary, supplementary and/or antagonistic, directly affecting the risk of ill-health, disability and premature death. Together, they can be considered “life-course health opportunity architecture” (11). They apply across the social gradient in health, meaning that the less advantage a person has, the greater their risk of poor health outcomes (11, 12). As many of these determinants already cause disparities in health and well-being in early-life, a life-course approach is necessary. Unlike a disease-oriented approach, which focuses on interventions for a single condition, a life-course approach considers the critical stages, transitions, and settings where large differences can be made in promoting or restoring health (13). Recognition of the need for a life-course perspective on prevention is operationalised through reference to well-becoming as well as well-being [see Figure 1; (6)].

All this is with the underlying acknowledgement that politics plays a role in setting and maintaining a social contract in a population with respect to our life-course health opportunity architecture. We can think of the social contract as the relationship between society and our personal responsibility. Shafik (14) poses the following questions, “What does society owe each of us? And what do we owe in return?” The above does not detract from the requirement for people to co-produce their own health and prevent avoidable ill-health, but the extent to which they can do this is determined by the life-course health opportunity architecture of the society in which they live, referred to above (11). Though much ill-health, disability and premature mortality is preventable, most people in any population will develop illnesses that need treating and turn, as part of the social contract, to their health care system to which they will have most likely contributed to financially (e.g., through taxation or social insurance over their lifetime).

Approximately 75% of all diseases worldwide and 90% of all deaths in Europe can be attributed to preventable NCDs (15, 16). This costs European taxpayers €700 billion annually, equivalent to about 50% of the €1.5 trillion (6–13% of GDP) European Union (EU) governments spend each year on health care (17, 18).

The increasing incidence and prevalence of preventable diseases and widening inequalities across Europe strongly indicate that European health systems are under strain and unable to meet the demands of increasing incidence and prevalence of preventable diseases, widening inequalities, ageing populations, and the digital and green transition. The year 2023 marked the 45^th^ anniversary of the historic Declaration of Alma-Ata, which endorsed a focus on primary health care, defined as a set of basic care services provided outside of the hospital sector, as the key to achieving improved health outcomes throughout the world (19). Yet, 45 years later, countries are still trying to shift emphasis from hospital- and treatment-based care to primary care as a means to promoting health promotion and preventing avoidable ill-health, disability and premature mortality (20). Among OECD countries, for example, on average less than 3% of health care budgets are spent on health promotion and disease prevention (21). Hospitals are the largest providers of health care, accounting for close to two-fifths (37.4%) of all health care expenditure in the EU in 2020. Providers of ambulatory health care (24.8%) and retailers and other providers of medical goods (16.7%) were the second and third largest providers of healthcare in expenditure terms (18). Though the need for prevention and an imbalance in spending between treatment and prevention was identified a long time ago (22), the slow shift from treatment- to prevention-base models of care is likely due to the complexity and timescales of returns from health promotion and disease prevention. Prevention requires action and investment from many actors throughout the life-course, often across long time horizons.

Effective primary health care models unite population public health services with individual primary care and encourage action across sectors to address the determinants of health (23). Boosting primary health care in communities can also be helpful for improving population health. In the United Kingdom (UK), for example, the King’s Fund has highlighted that much of local authority spending has the potential to promote health and prevent premature mortality (24). Likewise, municipalities have this potential in Scandinavian countries. This requires the potential for shared budgets and collective decision-making. The hospital-centric model consolidated across Europe in the 1980s and 1990s, however, retains political and emotional capital. Whilst strides have been made in strengthening primary care and integrated care models, there is still a cultural and political adherence to the hospital- and treatment-based model of care (25, 26).

The challenge is to transit over time to newer, broader and more integrated care models that encourage cross-sector collaboration to address determinants of health and attract innovative investment from across the public, private and third sectors. This is not a simple question of reducing the resources devoted to curative services (making up 53% of healthcare expenditure in the EU), which are under huge stress in most countries (18). There is a need to invest in new primary and secondary preventative models while maintaining the resources in care services. While not a doubling of expenditure—public health spending as defined is typically only small relative to that used for care, and never will be of comparable size—it will require an expansion of spending while new models are developed and tested. In addition to reallocating some wasted resources in our current care models, there is also an “invest to disinvest” situation ahead for population healthcare systems, which is likely to be controversial and difficult to implement.

### Investing in health promotion and disease prevention through smart capacitating investment (SCI)

To catalyse and drive a shift to prevention-based models of care, we launched the Invest4Health (I4H) project in 2023 to demonstrate that “smart capacitating investment” (SCI) could support a more efficient use of overall resources to achieve the dual aims of providing necessary curative care for those who are ill while maximising the returns on investment in health promotion and disease prevention to achieve more optimal health outcomes.

The term “smart capacitating investment” (SCI) was first introduced as part of Working Group 1 of a High-Level Task Force chaired by Romano Prodi, economist and 10th president of the European Commission (27). The term SCI built on previous work around capacitating social investment (28), and was seen by the Working Group as a means to boost longer-term investment in education and lifelong learning, health and long-term care, and affordable housing. Fundamentally, it was agreed that a new financial approach was needed to escape the underinvestment trap facing social infrastructure, not just within each sector but between sectors as we shift to integrating services in an ageing society. Before this, the term “capacitating investment” had been associated with a shift in social welfare policy from the palliation of harm to the prevention of harm across the life-course (28–30).

In the context of public health, health promotion, and disease prevention, we define “investment” as an increase in a particular capital stock, often the use of funds. The stock and investment in it can encompass non-financial or intangible resources (i.e., not just financial). It is likely that many preventative measures will have limited fixed assets—but there is still an up-front investment commitment before subsequent benefits flow. “Financing” refers to the source of funds for investment and operations. We do not expect these terms to be consistently used in the literature where the term investing might be used merely to indicate spending approved budgets. When used as such, there will be a grey zone between funding investments proper and routine financing over time. SCI-compatible business and finance models, while still being explored and developed, could potentially support improved delivery of care and help capacitate other health-promoting expenditures (e.g., housing, education, transport and access to nature) to achieve better and more equitable health outcomes.

**Figure 2:**
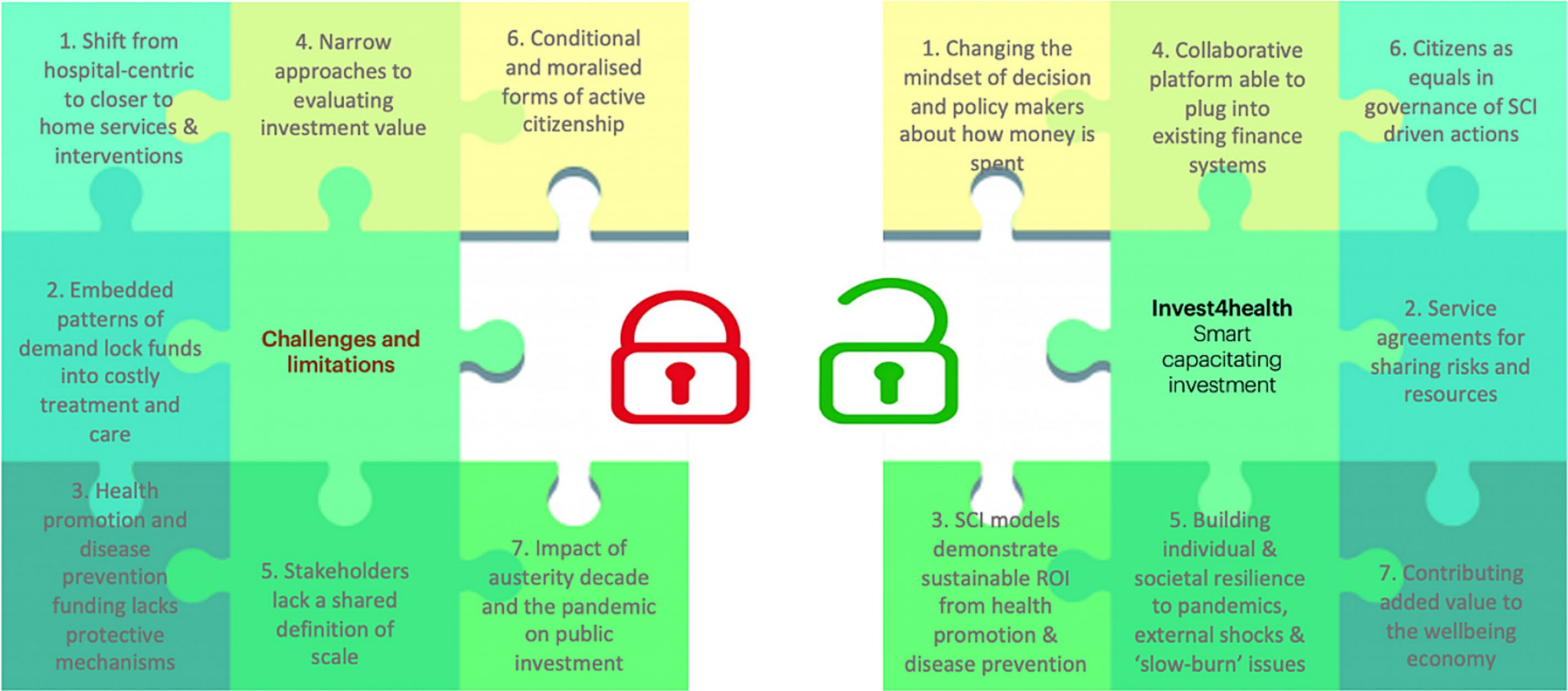
Using SCI to address current challenges and limitations in financing health promotion and disease prevention (Joanna Lane: May 2022, revised July 2023).

Figure 2 shows the shift from hospital-based health care systems to SCI in health promotion and disease prevention. The challenges and limitations shown in Figure 2 portray the starting point for the I4H project. For the last 30 years the focus has been on short-term easing of pressures due to continuing financial instabilities while the dominant care model embedded patterns of demand that locked funds into increasingly costly treatment and care. We argue that greater investment in prevention is needed, not only in health systems but between sectors as well. This can be thought of as integrated cross-sectoral prevention. The I4H project proposes that the seven factors related to SCI in Figure 2 need to be addressed to overcome the challenges and limitations.

Our cross-disciplinary and cross-country approach gives sensitivity to cultural and market differences, and an awareness of the different stages of maturity of the local health and well-being economies. The I4H consortium will develop innovative SCI models and associated tools for collaboration and investment at scale and across sectors that generates sustainable returns and localised benefits. SCI for health promotion and disease prevention is likely to need multi-sector stakeholders (e.g., at local authority or municipal level) and can take place at national, regional as well as local and community levels. SCI will create a positive impact through not only designing and validating new SCI-compatible financing and business models but also through generating expertise, networks, and other types of support to help stakeholders reach their full potential in enhancing health promotion and disease prevention.

## Methods

**Figure 3:**
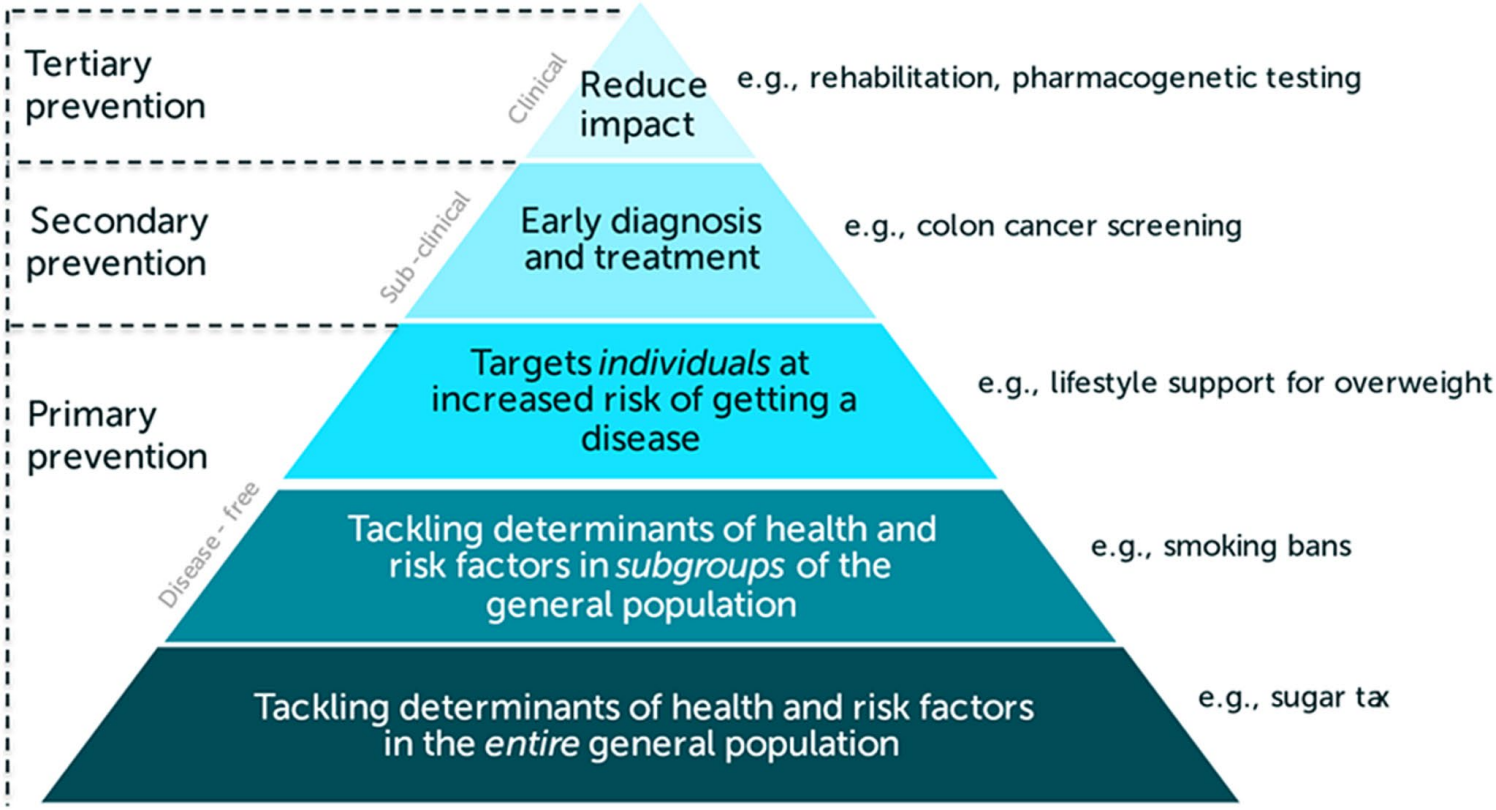
Prevention pyramid (50).

The I4H project focuses on SCI in public health. Two key areas of public health are health promotion and disease prevention, which are not mutually exclusive. Health promotion is the process of enabling people to co-produce and improve their own health through their own actions (31). Together with the environments in which people live, this approach addresses the root causes of ill-health. Figure 3 summarises our understanding of disease prevention in terms of primary, secondary, and tertiary levels of prevention in the prevention pyramid (with primary and secondary prevention yielding the greatest population health benefits) (32). On the right-hand side, examples of preventative interventions are listed to indicate that we do not only consider interventions in the health sector. We will also consider interventions in other sectors, such as fiscal interventions, housing, education, transportation, and planetary health that may impact human health. These could include intersectoral interventions where two or more sectors work together to deliver multi-sectoral outcomes, including health benefits (e.g., older adult care programs to support ageing in place as an example of tertiary prevention). The above has been referred to as “primordial prevention” (33).

### Objectives

**Figure 4:**
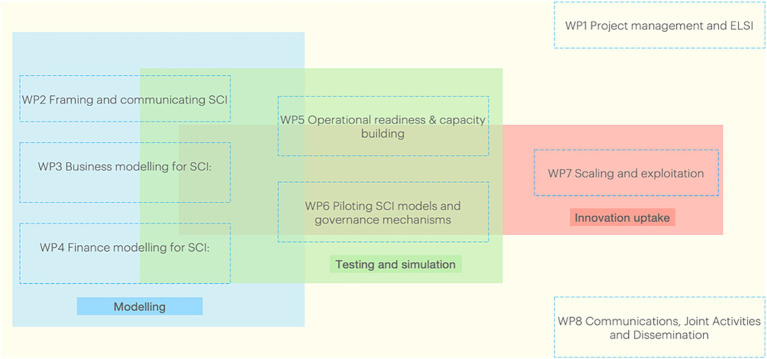
The eight work packages (WPs) of the Invest4Health (I4H) project.

The I4H project has five overarching objectives, which bring its eight work packages (WPs) together (see Figure 4). The first objective is to draw on available evidence from scientific and grey literature to help strengthen our understanding of, and insight into, how the SCI concept can be framed and communicated (WP2). The second objective is to assess and enhance organisational readiness for testing SCI models in regional testbeds (Sweden, Spain, Germany and Wales UK) (WP5, WP6). The third objective is to develop, adapt and test business models that are compatible with SCI (WP3, WP6). The fourth objective is to develop and test novel finance models which align both with the business models and with pre-defined contingencies for delivering SCI—identifying by whom they are financeable and defining a framework for measuring their impact across different sectors (WP4, WP6). This workstream will include examination of appropriate payment mechanisms, adapted to the investment sources. The fifth objective is to develop and test a prototype collaborative platform for co-governing SCI in health promotion and disease prevention (WP3, WP6, WP7). Hence, the content of WPs 2–4 is the scientific and novel heart of the project, enabled by the remaining WPs particularly for implementation and scalability.

### Framing and communicating SCI

In WP2 we will develop a conceptual framework to systematically describe SCI. This will be based on a scoping review of the scientific literature as well as the grey literature and a realist synthesis that uses a logic model approach to identify what works, for who and in what setting (34, 35). We will explore the possibility to develop a typology of SCIs that is linked to the levels of the prevention pyramid (as shown in Figure 3). We will also create a self-assessment tool for the regional testbeds to assess their readiness to apply SCI in-situ.

### SCI-compatible business and finance models

In WP3, we will study and compare existing business models (36) for health promotion and disease prevention. We will explore SCIs within local testbeds, economic and social contexts (WP6). The overarching business models will aim to integrate the conceptual framework of SCI (WP2) with the proposed finance models (WP4). A central feature of these business models will be to situate at their core, collaborative spaces and/or digital platforms. Collaborative spaces will enable multi-stakeholder engagements following the Quadruple Helix Model (37), to facilitate stakeholder involvement in the co-production and co-governance of SCI interventions. Citizen engagement and involvement, in particular, will be key to help address equity and diversity considerations in SCI implementation. The aim of the digital platform will be to explore digital architectures that may allow the combination and sharing of health and social care data and financial data to monitor use of resources, other costs and potential savings securely, so that they can be accessible for, among other things, effective monitoring and evaluation, as well as wider health promotion and disease prevention research. The overall governance of the collaborative spaces and platform can help to build trust, develop incentives, and better understand the replication and scaling opportunities of SCI models.

In WP4, the SCI conceptual framework of WP2 will be used as a starting point to develop investment models where actors beyond the usual funders of public health (defined at the level of each country or region concerned) are incentivised to invest in health promotion and disease prevention programmes. The mechanisms of channelling funds between investors, commissioners, management organisations and service providers (health financing models) will be tailored to the local context of each testbed, in accordance with the preferences expressed by their stakeholders.

Our approach to identifying and developing business and finance models (WP3 and WP4, respectively) to support SCI for health promotion and disease prevention is twofold: (1) identify theoretical models that may be applicable and anchored, even in part, to our testbeds, and (2) study existing models and develop a comparative analysis of factors and system-level dynamics that may allow us to offer template designs for operational, replicable and sustainable preventive (eco)systems. A part of this system-level comparative analysis is to identify and include factors beyond the economic, such as shared values, ethical norms, independent and collective incentives and general business principles of the diverse stakeholders involved in the SCI-driven collaborative models. Understanding these factors will be consequential to designing new collaborative models. In doing this, we will primarily focus on exploring novel ways of sharing public sector expenditure across sectors responsible for influencing determinants of health (health, housing as well as non-public sector parties). Secondly, we will be cognizant of the ongoing exogenous policy landscape affecting gains and losses to the public purse through the regulation or incentivisation of parties bearing some responsibility for the commercial determinants of health. These are largely outwith the scope of this project.

We will rely on data collected through interviews from experts and stakeholders, with emphasis on testbed regions. This data will be complementary to the knowledge generated from the scoping literature review (WP2, WP4). As an iterative process, routine workshops with testbeds will contribute to stakeholder and ecosystem mapping, and interview guide preparation.

### An evaluation framework for SCI

A framework for a cross-sectoral evaluation of SCI will be developed and applied to assess the value for the invested resources of the four testbeds (WP4). The framework will be based on multi-criteria decision analysis (MCDA) and will incorporate the relative preferences of stakeholders for the benefits of SCI. MCDA is a decision-making methodology that involves assessing and evaluating multiple criteria or alternatives (e.g., different stakeholders’ preferences) to make decisions (38). Depending on data availability and possibility for a prospective study design, different methodological approaches will be used in order to facilitate differences in evaluation opportunities between the testbeds and provide a reach demonstration of applying the framework to assess the benefits (of any kind) and opportunity costs across different sectors of public health interventions in the future. The self-assessment tool to assess testbeds’ readiness for SCI, developed in WP2, will guide the choice of outcome measures that will be used. WP4 methodology will include (1) a realist synthesis based on the findings of the scoping review of WP2; (2) expert interviews with key informants having experience in social outcomes financing and related models, and (3) testbed interviews and workshops for making explicit the preferences of local stakeholders.

### SCI capacity building

In WP5 we will assess regional readiness and, where feasible, support regional/local actors to prioritise health promotion and disease prevention programmes and initiate change in how they are financed. Based on work in WP2, as well as qualitative interviews and observation, we will assess operational challenges that relate to applying SCI models. Subsequently, we will support organisations within the testbeds to construct the structures and processes needed—that is, collaborative spaces, data platforms, general public involvement via participatory governance, SCI business models (from WP3) and SCI finance models (from WP4). These will enable continuous management of interactions between stakeholders in financial decision-making and ensure knowledge development over time. To build and retain operational memory of implementing SCI models, we will synthesise findings from WP2 to WP6 into a series of capacity building and training exercises for decision- and policy-makers. They will target the four pilot testbeds (Sweden, Spain, Germany and Wales UK) initially and then be extended to new scale-up locations (WP7). We will also engage in advocacy activities to enhance awareness and adoption of SCI models for health promotion and disease prevention. In collaboration with WP2, we will conduct foresight exercises within testbeds with decision- and policy-makers, investors, implementers, and the general public, with the aim of developing a shared vision for equitably improving health and well-being. Subsequent evaluation and learning, closely tied with WP4, WP6 and WP7, will be converted into a series of policy and process recommendations to create more favourable environments for innovation and cross-sectoral collaboration.

### Piloting SCI models and governance mechanisms in regional testbeds

In the testbeds (Sweden, Spain, Germany and Wales UK) we will undertake piloting and simulation of business and finance models and alternative governance mechanisms for SCI in health promotion and disease prevention (WP6). The four initial regions represent a range of health systems (see Table 1).

**Table 1:** The four testbeds in the Invest4Health (I4H) project.

Region and country	Population	Health system	I4H focus
Galicia, Spain	2.7 million	Universal single payer (i.e., National Health Service [NHS])	Active and healthy ageing
Nordrhein Westfalen, Germany	17.9 million	Universal multi-payer (i.e., statutory health insurance [SHI])	Health in the home office environment
Skåne, Sweden	1.7 million	Universal and decentralised health care	Life-course interventions for children’s and young adults’ health and well-being
West Wales, UK	383,000	Universal health care (i.e., NHS)	Social prescribing to promote co-production of better health

### Scaling and capitalisation

In later stages of the project, WP7 will leverage social franchising to develop a plan to capitalise on the project results to achieve scalability for SCI models across different contexts, considering the heterogeneity of local social needs and local health and well-being economy structures. Social franchising is defined as a system of contractual relationships “usually run by a non-governmental organization which uses the structure of a commercial franchise to achieve social goals” [(39), p. 129]. It provides opportunities for rapid and sustainable scaling of social impacts whilst offering flexibility for both the franchise and franchisees, making it a suitable approach for adapting SCI to different local and regional contexts.

Building on our learning from developing, experimenting and evaluating SCI-compatible business and finance models in regional testbeds (WP3, WP4, WP6), integrating them with collaborative spaces and digital platforms for co-production and co-governance with decision-makers and the general public (WP3), we will develop a roadmap for implementing SCI in different local-to-regional settings. This roadmap will be accompanied by the stakeholder resources and training developed in WP5. The final phase of the I4H project will involve an open call to offer our SCI package to new testbeds across Europe and explore social franchising as a vehicle for SCI implementation at scale.

### Plan for in-project learning and knowledge exchange

The Common Architecture Reference Group (CARG) within I4H is a multi-disciplinary learning forum that collates and reviews the knowledge threads generated from activities and discussions within and between the WPs as we explore, test and strengthen the I4H concepts and related knowledge building. It is designed to ensure that terms are used consistently within the I4H project, and in ways that are recognisable to public policy circles across Europe and beyond. In particular, it will help us map the architectural framework (critical elements and relationships) that are key to SCI in public health. The framework will adapt as the project proceeds and our understanding of how to make SCI work deepens. This is a process of collective sense-making (40) that projects and organisations rarely make time for. Above are our preliminary plans for academic outputs from the eight WPs (see Table 2). There will be a great deal of cross-WP integration in these outputs.

**Table 2:** Early publications by work package.

Work package (WP)	Outputs
WP1	Common Architecture Reference Group paper (with WP8 and WP2-WP7).
WP2	Overarching protocol paper. Rapid review protocol and published paper. The aim of this scoping review is to clarify the concept of SCIs in public health, including key defining characteristics, in the context of individual, community and population health. This will be undertaken by scoping the academic and grey literature relating to existing innovative financial and non-financial investments for health promotion and disease prevention. Rapid realist review protocol and published realist synthesis paper. The aim of this realist review is to explore for whom, in what circumstances or contexts, and how the concept of SCIs can influence individual, community and population health. As well as identifying potential barriers to successful SCI implementation. The realist review will develop an overarching programme theory for SCIs using evidence from the scoping review before exploring wider context conditions conducive to successful/unsuccessful SCI implementation.
WP3	SCI compatible business models, mapping the process of Business Modelling for SCI, as a longitudinal study.
WP4	SCI compatible finance and investment models.
WP5	Assessing readiness and operational challenges in testbeds applying different SCI-models, especially focusing on mechanisms that support flexibility and adaptability.
WP6	Assessing the developmental process of the four testbeds using logic models and problem tree approaches. Assessing potential additional testbeds and their applicability for SCI-models.
WP7	Capitalisation plan for scaling up SCI through Social Franchising.
WP8	Common Architecture Reference Group paper (with WP1 and WP2-WP7).

## Discussion

### Allocative and technical efficiency considerations

The I4H project will largely address questions of technical efficiency, including the mobilisation of finances to enable SCI in what are, it is to be assumed, agreed goals of allocative efficiency (e.g., the prevention of avoidable ill-health, disability and premature mortality) (41, 42). We are looking to find the best models of investment in health promotion and disease prevention. We already have a plenitude of health promotion and evidence-based prevention policies and programmes that need investment, for example, the World Health Organization’s (WHO) “Tackling NCDs: Best buys” (43, 44). What is lacking is resources, which we hope to resolve by applying SCI models of finance. Given such innovative finance models, we aim to identify the incentives and nudges for various stakeholders in preventive ecosystems, as well as those outside of preventive ecosystems to help them see the importance of prevention, to engage in collaborative and collective action in keeping these systems operational and sustainable (with respect to nudge theory see (45)).

### Expertise across the I4H consortium

The I4H consortium brings together experts and actors from different backgrounds, disciplines, and with different health and economic perspectives, to explore and develop common ways forward. We aim to explore and develop SCI models and associated tools that have wide applicability across diverse cultures and health care systems across Europe. This includes differing social contracts relating to state and personal responsibility for health and well-being (14).

### Acknowledging life-course health opportunity architecture and the importance of sustainability as underpinning our I4H project

I4H will use frameworks of health economics analysis which are underpinned by modern neoclassical economic theory. We recognise the existing distribution of wealth and inequalities in wealth and hence life-course health opportunity architecture across European societies—and indeed the mixed economy underlying production structures relating to health and well-being of the population (11). We also acknowledge challenges to modern neoclassical economic concepts of the importance of “growth” through, for example, recognition of alternative paradigms such as Doughnut Economics (46), which advocate for greater policy focus on sustainability and well-being in the face of zero or declining economic growth within planetary limits and the need for a safe and just space for humanity (46). Of course, the foundations of these ideas lie in the work of Schumacher (47) in *Small is Beautiful: A Study of Economics as if People Mattered*. Most recently, they have been expressed by Victor (48) in *Escape from Overshoot: Economics for a Planet in Peril*. We aim to be explicit where we refer to resource allocation decision points that necessitate value judgement. These decisions will be made by competent regional stakeholders in the testbeds, and their preferences will be made as transparent as possible.

### Application of I4H principles in low-resource settings

Low-resource settings are typically characterised by a lack of funds to cover health care costs, at an individual or societal scale. In low-resource settings, the SCI approach to sharing risks and resources (facilitated by new business and financial models) offers a solution to address several factors that hold these locations back from sustainable development: financial shortages, human resource limitations, suboptimal healthcare service delivery, lack of knowledge, underdeveloped hard and soft infrastructure, and restricted social resources (49). There may also be reverse technology learning from these low-resource settings to European countries which we need to consider to avoid inherent “global North bias” as we progress through the I4H project. These low-resource settings exist in all six of the WHO regions although needs and circumstances differ. So, while we continue to develop and test the different elements of SCI in EU testbeds, it is clear that SCI might have wider relevance globally including, for example, the WHO initiative on making hospitals fit-for-purpose in primary care-oriented health systems.

### The opportunities provided by public and private partnerships

We are not advocating for health promotion and disease prevention services to be exclusively provided by means of public versus private versus public and private partnerships (PPP), but we do seek to extend and go beyond the current, strained sources of investment. We do so by examining (through our four testbeds) new ways to collaborate among existing, including previously disconnected, stakeholders across multiple sectors working towards one shared collective goal, and identifying finance and business models that can help to sustain these dynamics. These new mechanisms or approaches to collaboration can and should engage a wide range of actors with improving health equity as a key shared goal of their collective engagement.

### Influencing and shaping future legislation

In the I4H project we will develop and adapt models at regional and local levels, and when they are mature enough, potentially can involve national legislation in their scale-up.

### Challenges and risks

As an international research consortium, we acknowledge potential (external to the project) challenges and risks of cross-jurisdictional working, variation in health and well-being economies and the extent to which public sectors and markets play a role. Internal to the project challenges and risks can be categorised as theoretical, data access, governance, staffing and communication. Specific examples of how we will meet such challenges and risks are presented in Table 3.

**Table 3:** Specific examples of how we will meet challenges and risks in the Invest4Health project.

Risk type	General mitigation strategy	Specific I4H example
Theoretical	Several underlying principles are important to I4H—e.g. having a common understanding of ‘prevention’, SCI, etc. We are developing a Common Architecture Reference Group to consolidate multi-disciplinary knowledge in an open forum.	Developing a working definition for SCI to be used across all WPs. All subsequent work will need to be informed by standardised, agreed definitions.
Data access	Close working relationships with testbed partners (including testbed data managers) and continual dialogue to understand data requirements from the research team and accessibility of data on testbed side.	Accessing relevant data from testbed partners.
Governance	Detailed quality assurance, data management, ethical, legal and social implications and risk management led by a dedicated team in WP1 to provide essential project governance. Appointment of an international advisory board with wider stakeholder representation.	Potential timeline misalignments across WPs managed by overarching project governance and routine WP-specific and consortium wide meetings.
Staffing	We routinely revisit our staffing plan as a consortium to identify any staff turnover and will conduct continuation and succession planning of newly integrated staff if required. Person hours on the project agreement allows for some flexibility of named persons.	The potential for key staff leaving, particularly in the testbeds where organisational memory is important.
Communication and dissemination	Dedicated WP8 lead on inward and external communication and publications to ensure all outputs are of highest quality. Dedicated knowledge mobilisation and impact plans developed to steer outputs to appropriate audiences through multimedia outputs. We are developing a public and patient involvement group that will inform our dissemination to public/lay audiences.	Uncoordinated publication plan and undervalued impact planning.

## Conclusion and future research

The I4H project aims to incentivise a shift from hospital- and treatment-based care to SCI in health promotion and disease prevention at scale across multiple sectors to generate sustainable returns and localised benefits. We will develop and test SCI models and associated governance mechanisms and tools with decision- and policy-makers and citizens to facilitate cross-sector collaboration and innovative investment in health promotion and disease prevention, promoting co-production of better health with the population. Our planned deliverables will have wide generalisability across countries facing increasing pressures on health and social care systems and their allocated budgets. I4H also paves the way for further transdisciplinary research on SCI within public health more broadly.
